# Enhancing CAR T-cell therapies against solid tumors: Mechanisms and reversion of resistance

**DOI:** 10.3389/fimmu.2022.1053120

**Published:** 2022-12-08

**Authors:** Yue Qin, Guotai Xu

**Affiliations:** ^1^ National Institute of Biological Sciences, Beijing, China; ^2^ Tsinghua Institute of Multidisciplinary Biomedical Research, Tsinghua University, Beijing, China

**Keywords:** CAR T-cell, solid tumor, antigen viability, microenvironment, tumor infitration, combined therapy

## Abstract

Chimeric antigen receptor (CAR) T-cell therapy, belonging to adoptive immune cells therapy, utilizes engineered immunoreceptors to enhance tumor-specific killing. By now new generations of CAR T-cell therapies dramatically promote the effectiveness and robustness in leukemia cases. However, only a few CAR T-cell therapies gain FDA approval till now, which are applied to hematologic cancers. Targeting solid tumors through CAR T-cell therapies still faces many problems, such as tumor heterogeneity, antigen loss, infiltration inability and immunosuppressive micro-environment. Recent advances provide new insights about the mechanisms of CAR T-cell therapy resistance and give rise to potential reversal therapies. In this review, we mainly introduce existing barriers when treating solid tumors with CAR T-cells and discuss the methods to overcome these challenges.

## Introduction

Immune therapies are powerful for cancer treatment and, regardless of the drug types used (chemotherapy, radiotherapy or chemical compounds with specific targets), tumor regression usually involves participation of the immune system (1, 2). Immune checkpoint blockage (ICB) can unleash the inhibition of immune response and enhance antitumor effect (3, 4). However, the expression of immune checkpoint is not tumor unique and may result in strong side effects (5, 6). Furthermore, ICB usually could not trigger an effective response due to few tumor-infiltrated lymphocytes (7). Recent studies showed that loss of some genes may elevate PD-1/PD-L1 in tumor niche and, however, enhanced immune cell infiltration, while the overall result of such gene loss tends to be reduced tumor growth which indicates the power of immune cells (8–12).

**Figure 1:**
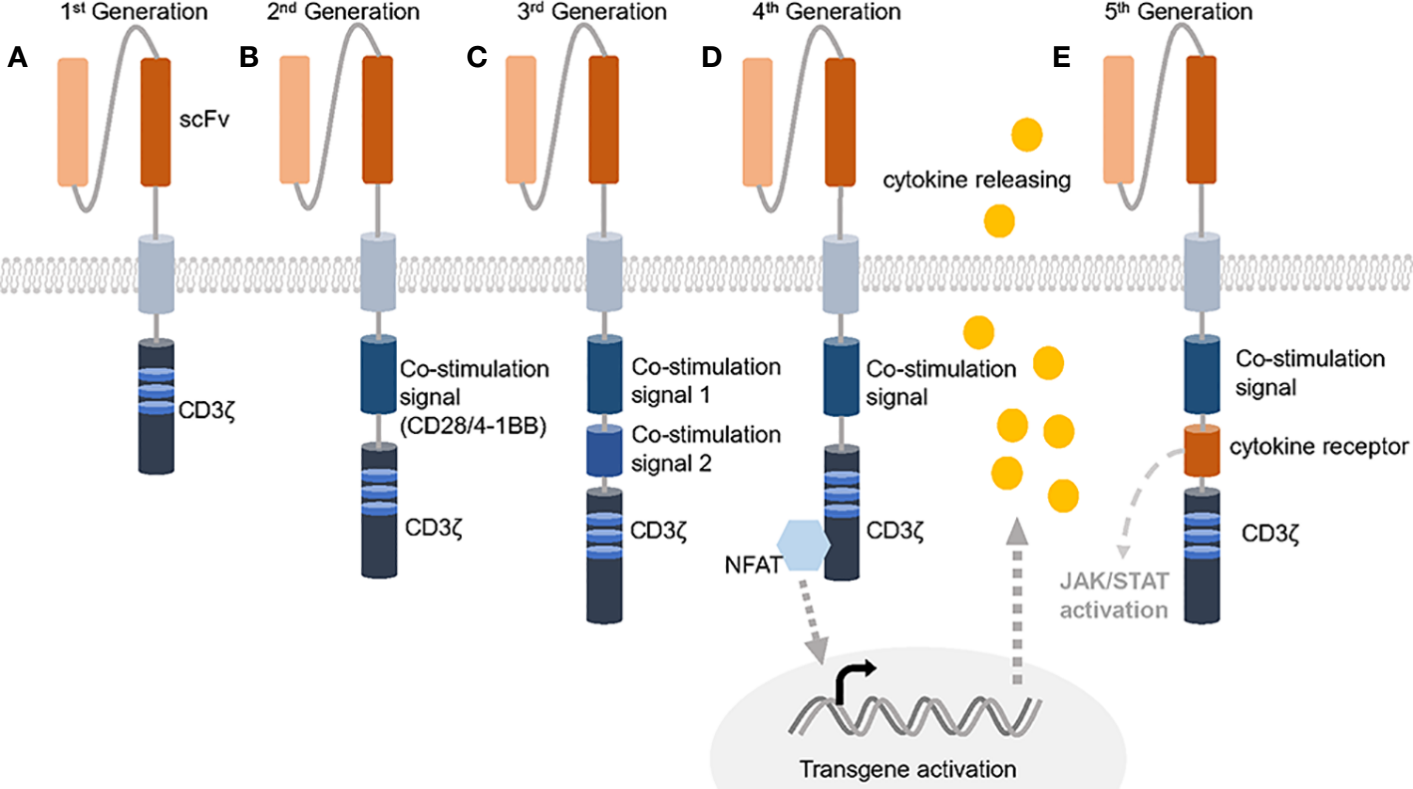
Five generations of CARs. **(A)** The 1st generation of CAR only contains the extracellular scFv and intracellular CD3ζ activation domine, which could not trigger enough active response. **(B)** 2^nd^ generation of CAR has an additional co-stimulation domain based on 1st generation and obtained success against leukemia. **(C)** 3rd generation of CAR increases the number of co-stimulation domain and evoke stronger stimulation after antigen binding. **(D, E)** 4^th^ generation of CAR is usually referred to TRUCKs that contains one more cytokine inducer compared with 2^nd^ CAR to promote cytokine-mediated killing. 5^th^ CAR can both activate the cytokine receptor dependent JAK/STAT signal and perform the normal function of a 2^nd^ CAR.

Proposed in 1989, CAR T-cells have evolved for three decades and usually employ the TCR activation intracellular domain fused with antibody variable domains to kill specific antigen bearing tumor cells (13, 14). Comparing with traditional adoptive cellular therapies that are composed of unmodified host T-cells, CAR T-cells become more sensitive and precise (15). Besides, many attempts gradually improve the effectiveness by utilizing different activation domains or combining costimulatory domains (**Figure 1**). The 1st generation of CAR contains single chain variable fragment (scFv) and a single CD3ζ subunit which is the intracellular part of CD3 complex that mediates the MHC-TCR signaling transduction to activate T-cells but showed poor efficacy, while adding one costimulatory signaling domain from CD28-CD3ζ or 4-1BB-CD3ζ is now commonly and successfully applied, called 2nd CAR. Furthermore, 3rd CARs that combined both CD28 and 4-1BB give the T cell stronger activation signals (16, 17). The FDA has approved six 2^nd^ CAR T-cell therapies since 2017 as the result of positive clinical cases for anti-blood cancer treatment including acute lymphoblastic leukemia (ALL), non-Hodgkin lymphoma (NHL), follicular lymphoma, mantle cell lymphoma (MCL) and multiple myeloma (18–21).

Despite the encouraging results in leukemia, many CAR T-cell related clinical trials are still going on for solid tumors (**Table 1**) (22). Some shows positive response and demonstrate the safety of CAR T-cells. In 2016, the early successful case of CAR T-cells targeting solid tumors is reported and proved the feasibility (23). However, others face many problems such as antigen loss, T-cell exclusion and defects in T-cell infiltration (24–27). As engineered cells, CAR T-cells can be further improved to tackle the specific challenges (28, 29). Now, the 4th and the 5th generation of CARs are born as well as some other modifications on CARs like the dual-antigen targeting CARs that possess dual specificity and dual co-stimulation signal (30, 31). Such CARs help T-cells to sustain longer and act wider. Considering the complexity of solid tumor treatment and abundant state-of-the-art strategies, we divide this review into six topics containing targeting solid tumor antigen variability, enhancing tumor infiltration and T-cell activation-rest cycling, dealing with immunosuppressive microenvironment, controlling CAR T-cell epigenetics changes, choosing the suitable chassis for CAR T-cell production and utilizing combined therapies. In the review, we plan to explain the resistance mechanism and recent efforts to overcome the barriers.

**Table 1:** Current ongoing CAR T therapy clinical trials in solid tumors.

Target	Clinical trial number	Treatment	Cancer types	Region
MSLN	NCT05373147	αPD1-MSLN CAR-T	Solid Tumor	China
	NCT05531708	MSLN-CAR T	Mesothelin-positive Advanced Refractory Solid Tumors	China
	NCT05166070	MSLN-CAR T	Solid Tumor	
	NCT04489862	αPD1-MSLN-CAR T	Non-small-cell Lung Cancer	China
			Mesothelioma	
	NCT05141253	MSLN-CAR T	MSLN-positive solid tumors	China
	NCT04981691	MSLN-CAR T	Refractory Malignant Solid Neoplasm	China
			
Claudin 18.2	NCT05583201	Claudin 18.2 CAR-T	Gastric Cancer	China
			Pancreatic Cancer	
			Solid Tumor	
	NCT05472857	Claudin 18.2 CAR-T	Gastric Cancer	China
			Pancreatic Cancer	
			Advanced Ovarian Carcinoma	
			Gastroesophageal Junction Adenocarcinoma	
	NCT03874897	Claudin 18.2 CAR-T	Advanced Solid Tumor	china
		PD-1 Monoclonal Antibody		
		Chemotherapy		
	NCT04467853	Claudin 18.2 CAR-T	Solid Tumors	China
	NCT05275062	Claudin 18.2 CAR-T	Advanced Solid Tumors	China
			Gastric Cancer	
			Esophagogastric Junction Cancer	
			Pancreatic Cancer	
	NCT05199519	Claudin 18.2 CAR-T	Solid Tumor	China
	NCT04581473	Claudin 18.2 CAR-T	Gastric Adenocarcinoma	China
			Pancreatic Cancer	
			Gastroesophageal Junction Adenocarcinoma	
CLDN6	NCT04503278	CLDN6 CAR-T	Solid Tumor	Germany
NKG2D	NCT05583201	NKG2D/CLDN18.2 CAR-T	Gastric Cancer	China
			Pancreatic Cancer	
			Solid Tumor	
	NCT05382377	NKG2D CAR-T	CRC	China
			Solid Tumor	
	NCT05131763	NKG2D CAR-T	Hepatocellular Carcinoma	China
			Glioblastoma	
			Medulloblastoma	
			Colon Cancer	
GD2	NCT05437315	GD2/PSMA CAR-T	Solid Tumor	China
	NCT03373097	GD2 CAR-T	Neuroblastoma	Italy
			Neuroblastoma Recurrent	
			GD2-positive Solid Tumors	
			(and 3 more...)	
	NCT01822652	GD2 CAR-T	Neuroblastoma	United States
		Drug: Cytoxan		
Nectin4/FAP	NCT03932565	Nectin4/FAP CAR-T	Nectin4-positive Advanced Malignant Solid Tumor	China
CD22	NCT04556669	aPD-L1 armored CD22 CAR-T	Solid Tumor, Adult	China
			Cervical Cancer	
			Sarcoma	
			NSCLC	
CD70	NCT05518253	CD70 CAR-T	Metastatic Tumor	China
			Advanced Solid Tumor	
			Renal Cell Carcinoma	
			(and 2 more...)	
	NCT05420545	CD70 CAR-T	Metastatic Tumor	China
			Advanced Solid Tumor	
			Renal Cell Carcinoma	
			(and 2 more...)	
	NCT05468190	CD70 CAR-T	Metastatic Tumor	China
			Advanced Solid Tumor	
			Renal Cell Carcinoma	
			(and 2 more...)	
HER-2	NCT04650451	HER-2 CAR-T	HER-2 Gene Amplification	United States
			HER2-positive Gastric Cancer	
			HER2-positive Breast Cancer	
			(and 2 more...)	
	NCT03740256	CAdVEC	Bladder Cancer	United States
		HER-2 CAR-T	Head and Neck Squamous Cell Carcinoma	
			Cancer of the Salivary Gland	
			(and 7 more...)	
	NCT04511871	HER-2 CAR-T	Solid Tumor	China
			Gastric Cancer	
			Breast Cancer	
			(and 2 more...)	
	NCT03618381	CD19-HER2 CAR-T	Pediatric Solid Tumor	United States
		EGFR CAR-T	Germ Cell Tumor	
			Retinoblastoma	
			(and 13 more...)	
EGFR	NCT04483778	B7H3-EGFR CAR-T	Pediatric Solid Tumor	United States
		CD19-HER2 CAR-T	Germ Cell Tumor	
			Retinoblastoma	
			(and 14 more...)	
	NCT04976218	EGFR CAR-T	Solid Tumor, Adult	China
			EGFR Overexpression	
	NCT05341492	EGFR/B7H3 CAR-T	EGFR/ B7H3-positive Advanced Lung Cancer	China
			EGFR/ B7H3-positive Advanced Triple-negative Breast Cancer	
	NCT03618381	CD19-HER2 CAR-T	Pediatric Solid Tumor	United States
		EGFR CAR-T	Germ Cell Tumor	
			Retinoblastoma	
			(and 13 more...)	
VEGFR1	NCT05477927	VEGFR1/PD-L1 CAR-T	Malignant Peritoneal Effusion	China
			Malignant Ascites	
			Serous Cavity Metastatises	
EpCAM	NCT02915445	EpCAM CAR-T	Malignant Neoplasm of Nasopharynx TNM Staging Distant Metastasis (M)	China
			Breast Cancer Recurrent	
			Gastric Cancer With Metastasis	
CEA	NCT05415475	CEA CAR-T	Colorectal Cancer	China
			Esophageal Cancer	
			Stomach Cancer	
			(and 3 more...)	
	NCT05396300	CEA CAR-T	Colorectal Cancer	China
			Esophageal Cancer	
			Stomach Cancer	
			(and 3 more...)	
	NCT05538195	CEA CAR-T	Gastric Cancer	China
			Colon Cancer	
			Rectal Cancer	
			(and 2 more...)	
	NCT04348643	CEA CAR-T	Solid Tumor	China
			Lung Cancer	
			Colorectal Cancer	
			(and 4 more...)	
B7-H3	NCT04897321	B7-H3 CAR-T	Pediatric Solid Tumor	United States
		Cyclophosphamide	Osteosarcoma	
		MESNA	Rhabdomyosarcoma	
		Fludarabine	(and 13 more...)	
	NCT05341492	GFR/B7-H3 CAR-T	EGFR/ B7H3-positive Advanced Lung Cancer	China
			EGFR/ B7H3-positive Advanced Triple-negative Breast Cancer	
	NCT04483778	B7H3-EGFR CAR-T	Pediatric Solid Tumor	United States
		CD19-HER2 CAR-T	Germ Cell Tumor	
			Retinoblastoma	
			(and 14 more...)	
	NCT05190185	B7-H3 CAR-T	Malignant Melanoma	China
			Lung Cancer	
			Colorectal Cancer	
Glypican-3	NCT04377932	Glypican-3 CAR-T	Liver Cancer	United States
		Cytoxan	Rhabdomyosarcoma	
		Fludara	Malignant Rhabdoid Tumor	
			(and 3 more...)	
	NCT02932956	Glypican-3 CAR-T	Liver Cancer	United States
		Cytoxan		
		Fludara		
ROR2	NCT03960060	ROR2 CAR-T	Solid Tumor	China
			Soft Tissue Sarcoma	
			Gastric Cancer	
			(and 2 more...)	
ROR1	NCT05274451	ROR1 CAR-T	Triple Negative Breast Cancer	United States
			TNBC - Triple-Negative Breast Cancer	
			Non-small Cell Lung Cancer	
			(and 12 more...)	
MUC1	NCT05239143	P-MUC1C-ALLO1 CAR-T	Breast Cancer	United States
		Rimiducid	Ovarian Cancer	
			Non Small Cell Lung Cancer	
			(and 6 more...)	
	NCT04025216	TnMUC1 CAR-T	Non-Small Cell Lung Cancer	United States
		Cyclophosphamide	Ovarian Cancer	
		Fludarabine	Fallopian Tube Cancer	
			(and 3 more...)	
MUC16	NCT02498912	MUC16 CAR-T	Solid Tumors	United States
PSCA	NCT02744287	PSCA CAR-T	Metastatic Castration-resistant Prostate Cancer	United States
			Metastatic Prostate Cancer	
GUCY2C	NCT05287165	GUCY2C CAR-T	Advanced Solid Tumors	China
			Digestive System Neoplasms	
			Pancreatic Cancer Resectable	
			Colorectal (Colon or Rectal) Cancer	
GPC3	NCT05120271	GPC3 CAR-T	Hepatocellular Carcinoma	United States
			Squamous Cell Carcinoma of the Lung	
			Merkel Cell Carcinoma	
			Myxoid/Round Cell Liposarcoma	

## Targeting solid tumor antigen variability

In fact, TCR T-cells against NY-ESO-1 positive synovial cell sarcoma and melanoma showed great potential in 2010 even before the first successful case that CD19 CAR T-cell cured an Acute Lymphocytic Leukemia (ALL) patient in 2013, which indicated the anti-solid tumor efficacy of engineered T-cell therapies (32). CAR-engineered T-cells targeting IL13Rα2 induced regression of glioblastoma and 3/11 neuroblastoma patients achieved complete remission after GD2-CAR T-cells infusion (23, 33). However, the antigen heterogeneity is a major concern when treating tumor using the concept of precision oncology. Biallelic loss was found when multiple myeloma treated by anti-BCMA CAR T-cells while EGFRvIII-directed CAR T-cells infiltrated glioblastoma and induced on-target tumor killing but the tumor gradually acquired resistance due to antigen loss suggesting the difficulty to identify a unique tumor associated antigen (TAA) (34, 35). Though the leukemia also lacks TAAs, blood system has its tissue-specific marker such as CD19 that represents the B lymphocytes (36). Importantly, the eradicated B-cells can be reloaded by stem cells, which makes it suitable for CAR T-cell therapies.

**Figure 2:**
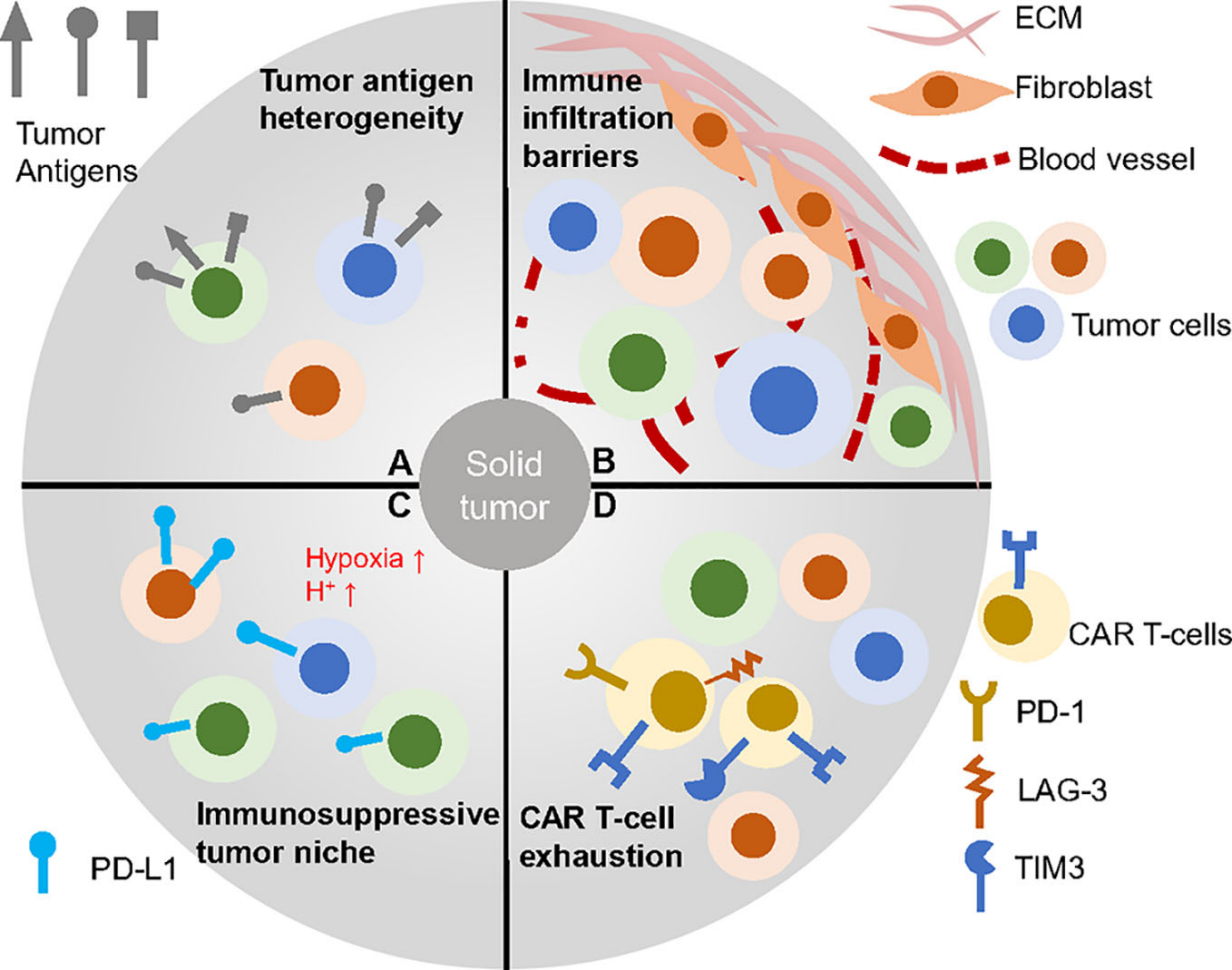
CAR T therapy faced challenges in solid tumors. **(A)** Solid tumor cells baring heterogeneous antigens are difficult to be targeted. **(B)** Complex tumor barriers inhibit immune infiltration. **(C)** Many factors contribution to the immunosuppressive environment such as low pH, hypoxia and immune check point blockage molecules. **(D)** CAR T-cells within the solid tumor often go exhausted, which impairs efficacy.

As for solid tumors, the tissues where they localized or derived are seldom capable of regeneration like the hematopoietic system (37). Thus, targets of CARs for solid tumors should be chosen more carefully and uniquely to avoid strong on-target, off-tumor cytotoxicity and properly for wider range of tumor cells to reduce antigen-loss effect (**Figure 2**). The ideal antigens would be the mutated oncogenes expressed on most tumor cell membrane but the proper neoantigens are rare and hard to determine (38, 39). B7-H3 is reported to be a checkpoint molecule highly expressed on brain tumors and antibody targeting B7-H3 results in potent antitumor activity (40). OR2H1 is an olfactory receptor widely expressed on ovarian cancers, non–small cell lung cancers as well as breast cancers but less detected on normal tissues. CAR T-cells targeting OR2H1 demonstrated antigen-specific cytotoxicity *in vitro* and *in vivo* suggesting it may be a promising target for epithelial cancers (41). Mesothelin (MSLN) is broadly overexpressed in many solid tumors including mesothelioma, lung adenocarcinomas and ovarian cancer while remains at a low level on mesothelial cells (42, 43). This target has been widely applied to immunotherapy, especially the CAR T therapy that resulted in successful clinical treatment (44). Beside epithelial cancer, pancreatic ductal adenocarcinoma (PDAC) is a highly lethal disease and many groups tried to find its specific targets. Using an antibody array screening, CD318, TSPAN8 and CD66c are identified to be proper PDAC antigen with limited expression off tumor (45). To validate the findings, CAR T-cells targeting these candidates are developed and exhibit anti-tumor response. CEACAM7 is another membrane protein strictly expressed on pancreas and colon and also proved to be an effective CAR T-cell target for PDAC (46). Recently, an B7-H3 CAR that has rear reactivity to B7-H3 -low tissues was developed and mediated clearance of abundant solid tumor xenografts (47).

In addition to broad spectrum tumor antigens, multi-specific CAR T-cells were manufactured to cope with antigen loss. GD2.28ζ/B7-H3.BB expressing CAR T-cells control SH-SY5Y derived tumors without escape compared with either GD2.28ζ or B7-H3.BB only CAR T-cells (31). Furthermore, tumors bearing low levels of TAG-72, which is an adenocarcinoma maker, can also be effectively eliminated through TAG-72 and CD47 dual-targeting CAR T-cells. Also, anti-CD19 and CD22 CARs demonstrated the efficacy in phase I clinical trials for B-ALL (48). One group even measured the trivalent CAR T-cell (HER2IL, 13Rα2 and EphA2) against glioblastoma and gained significantly positive results (49). Besides, CART.BiTE is a novel design with a CAR specific for EGFRvIII as well as a secreted EGFR-specific bispecific T-cell engager (BiTE). CART.BiTE cells can thus targeting the EGFRvIII-positive or EGFR-positive glioblastoma while activating bystander T cells, which effectively represses tumor growth (50). All the studies illustrate muti-targeting CAR could be an alternative way to overcome tumor heterogeneity in some solid and blood tumors.

Recruiting and activating other immune cells can also help to clear the tumor cells without CAR specific antigens. Such engineered CAR T-cells include proinflammatory molecule CD40 ligand or IL-7 and CCL19 or CCL5 and CXCL9 expressing cell to stimulate antigen-presenting cells (51–53). Despite the spontaneous antigen loss due to stress, T-cell trogocytosis can mediate antigen transfer from tumor cells to T-cells and cause T-cell fratricide (54). Though it is unknown how much such mechanism accounts for tumor escape of CAR T-cell treated patients, the study provides us a new dimension about tumor resistance to immune system.

## Enhancing tumor infiltration and T-cell activation-rest cycling

Solid tumors are enriched with extracellular matrix (ECM) and tumor associated fibroblasts and plenty of them are cold tumors, which means they lack complete blood vessels and proper conditions to recruit immune cells (55–57) (**Figure 2**). All of these characteristics block the infiltration of CAR T-cells and trap the intratumor immune cells. To tackle the problem, many research groups developed chemokine receptors bearing CAR T-cells that homes to the tumor bed as well as armed CAR T-cells against ECM. Chemokine receptor CCR2b mediates the CAR T-cells orientation to NSCLC tumors (58). CXCR1- or CXCR2-modified CAR T-cells also optimize the infiltration ability of T-cells in many tumor models (59). Except expressing chemokine receptors to facilitate homing, CAR T-cells are designed to seek specific antigens within tumors and therefore, tumor infiltrated CAR T-cells can act as lighthouses to recruit other immune cells, for example, IL-7 and CCL19 secreting CAR T-cells that attract other immune cells infiltration (52). Enzyme heparanase (HPSE) expressing CAR T-cells can effectively degrade ECM and enhance tumor infiltration with improved efficacy (60). VEGF is a cytokine important for angiogenesis of tumor cells. T-cells expressing anti-VEGF2 CARs inhibit multiple types of syngeneic tumors through increased immune infiltration (61). FAP-CAR T-cells are against fibroblast activation protein (FAP) expressing cancer-associated stromal cells. Although these cells are not intended to be direct tumor targeting, they destroy the tumor microenvironment and restrict solid tumor growth as well as desmoplasia (62).

Apart from the modification of CAR T-cells, combined therapies may facilitate CAR T-cells’ access to tumors. Docetaxel treatment upregulated the expression of CXCL11 and enhanced CD8^+^ T-cells recruitment (63). Low dose radiotherapies also can increase active T-cells in tumor microenvironment and normalize tumor blood vessels which may result from upregulation of several pro-angiogenic genes such as ANGPT2, TGFB2, FGF2 (64, 65). Intratumoral injection of CAR T-cells also directly exposes solid tumors to cytotoxicity and surmounts obstacles induced by tumor associated matrix and cells (43, 66). Prasad S. Adusumilli et al. found that regional delivery of M28z T-cells targeting MSLN showed superiority than the systemic route and Julia Tchou et al. proved the concept by intratumorally treating metastatic breast cancer with mRNA c-Met-CAR T-cell and showed improved tumor necroptosis (43, 67). One group compared regional delivery of CAR T-cells with intravenous injection in metastatic breast cancer and found improved effect using intracranial and intratumoral administration with complete tumor regression (68). Also, regional delivery of low dose anti-HER2 CAR T-cells induced durable regression of medulloblastoma (69). Though intratumor injection is a direct delivery method, this only helps CAR T-cells without other immune cells.

New materials can help the attraction of immune cells effectively. Recently, a kind of hydrogel without passive diffusion of cytokine was developed. Hydrogel containing cytokine and CAR T-cells can be injected near the tumor and create an inflammatory niche for improving CAR T- and immune cells efficacy (70). Similarly, fibrin gel accommodates CAR T-cells could effectively facilitate cell delivery either (71). Plenty of targets are discovered with the effect of enhanced immune cells infiltration. DDR1 is a collagen receptor whose expression is negative correlated with intratumoral T cells. DDR1 knockout tumors align collagen fibers and have more CD8^+^ T-cells. More importantly, tumor growth is significantly decreased without DDR1, from which CAR T-cell therapy can get benefit (72).

On the other hand, continuous antigen exposure changes the CAR T-cells to dysfunctional NK-like T-cells, which suggests that rest is vital for CAR T-cells’ normal cytotoxicity (73). Blood vessel is naturally born for cell traffic and thus when T-cells attack leukemia, they have chance to separate from the tumor antigen through blood flow and get rest (74, 75). In another way, CD8α-PILRα interactions mediated T-cell–myeloid cell cross-talk also maintains T-cell actively quiescence. Loss of the interactions may destroy naïve and memory CD8^+^ T-cell pools and results in poor T-cell function (76). However, solid tumors may lack the interaction but be enriched by antigen-CAR interaction. The CAR T-cells getting involved in these tumors could not have rest and therefore may become exhausted. In order to solve the challenge, inducible CARs and circuit CARs are developed for activation control and prove their efficacy (77). Synthetic intramembrane proteolysis receptors (SNIPRs) are a kind of inducible receptors based on the SynNotch module which is activated when binding with proper antigens (78). Such receptors can be coupled with CAR gene expression and only when the receptors counteract with the target antigen can the CARs be expressed on the cell membrane. This system provides a high signal-to-noise ratio for tumor specific killing. Another directed inducible CAR includes a protease controlling CAR degradation mechanism termed signal neutralization by an inhibitable protease (SNIP). Grazoprevir exposure causes protease inhibition and retains CAR function. The SNIP CAR shows significantly anti-tumor ability and more active T-cells without toxic effect (79). Ultrasound activation CAR provides precise T-cell activation that is restrict to the tumor site and may prevent always-on CAR T-cells as well as off-tumor cytotoxicity (80). Direct CAR signaling inhibitors such as dasatinib also help T-cells to rest and restores functionality (81).

## Dealing with immunosuppressive microenvironment

As a “living drug”, CAR T-cells need to be nursed (26, 82). Nevertheless, solid tumors could hardly provide a cozy home for the T-cells like in the blood. Immunosuppressive microenvironment is a common problem faced by all immune therapies (29, 83). One group recently demonstrated quiescent cancer cells created a niche to inactivate both T-cells and dendritic cells through increased expression of chemoresistance, hypoxia, and glycolysis related genes especially HIF1a suggesting the necessary to modify tumor niche (84).

To target the hypoxic tumor microenvironment, hypoxia-inducible CAR T-cells provide tumor cytotoxicity only within the low oxygen condition and avoid on-target, off-tumor effect (85–87). Normalization of tumor vascularity can reverse hypoxia and benefit CAR T-cell therapies (88, 89). VEGF and ANG-1/2 are two major factors impact tumor vascularity. Blocking VEGF by anti-VEGF antibodies can induce prolonged vessel normalization and overcome immune therapy resistance. However, some tumors develop drug resistance after such treatment. Combined VEGF and ANG2 blockade may confer the resistance and promote anti-cancer immunity (90, 91).

Apart from incomplete vasculature, solid tumor niche forces the expression of many immune checkpoint factors such as PD-1, CTLA-4, TIGIT and LAG-3 on T-cells (92). These immune checkpoints inhibit T-cell normal function and promote T-cell exhaustion. Therefore, studies demonstrated LAG-3 disruption or PD-1 deletion through genetic modifications induced robust antigen-specific antitumor activity (93, 94). Xiaojun Liu et al. utilized the PD-1/PD-L1 interaction and created a PD1-CD28 switch which activates T-cell upon binding to PD-L1. This design augments CAR T-cell cytokine secretion and antitumor activity (95). Newly founded checkpoint PTP1B also limits the CAR T-cell therapy indicating that the complexity of checkpoint family needs further study (96). Some chemicals within tumor microenvironment also inhibit immune function. Tumor necrosis releases potassium ions interfere with mTOR signaling causing impaired function and T-cells overexpressing potassium channel can reverse the tumor resistance (97). The pH value in TME is lower than normal tissue such as blood due to tumor-derived lactic acid, which drives enhanced negative regulatory signals and higher activation thresholds of T-cells (98). Lots of drugs are developed to neutralize acidic environment and their combination with CAR T-cells may control tumor growth (99).

Besides, cancer-associated fibroblasts (CAFs) are now well studied and heavily heterogeneous. Most ECM is the product of CAFs including immunosuppressive fibroblast activation protein (FAP). Deletion of these cells provides opportunities for immune therapies (100). In particular, adoptive transfer of FAP-CAR T-cells reduces tumor growth in a FAP-dependent fashion and can be combined with Ad.E7 antitumor vaccine (101). Usually, fibroblasts secreted CXCL12 or CCL2 are also immunosuppressive and thus blockage therapies can be considered as an assistance of FAP-CAR T-cells (102). Regardless of the fibroblasts, immunosuppressive cells components such as myeloid-derived suppressor cells (MDSCs), regulatory T-cells (Tregs), tumor-associated macrophages (TAMs) in the solid tumor also inhibiting antitumor immune responses. MDSCs and Tregs within microenvironment inhibit the CAR T-cells by producing TGF-β and interleukin-10 (IL-10) while Tregs also express checkpoint molecules such as PD-1 and CTLA4 (102–105). Furthermore, T_regs_ recruitment is associated with TAMs and interleukin-6 (IL-6) secreted by MDSCs recruits Th17 cells which strengthen the immunosuppressive effect. T_reg_ activation strongly inhibits CD8^+^ cell proliferation and it is reported that deleted the IL1R1^+^ T_reg_ could improve T-cell ability in solid tumors (106). Tumor cells also release many cytokines including CCL2, CXCL12 and CSF1 that attract TAMs to help tumor escape by producing IL-10 and also directly inhibiting CAR T-cells *via* PD-1-PD-L1 interaction (107). Thus, TAMs density is severely related to patients’ survival in solid tumors (108). FRβ is a protein universally expressed on M2 TAMs and studies demonstrated solid tumors pretreated with CAR T-cells targeting FRβ improves pro-inflammatory monocytes enrichment and tumor-specific CAR T-cells efficacy (107). This study in another way proved that the combination of two kinds of CAR T-cells may acquire better responses against solid tumors. Reasonably, strategies to directly inhibit TGFβ, IL-10 and cytokine signaling can reverse the immunosuppression situation either. For example, Inhibition of CSF-1 Receptor by PLX3397 shows promising results to assist adoptive cell therapy in melanoma model and TGF-β-receptor kinase inhibitor SD-208 shelters the ROR1-CAR T-cells from suppression (109, 110). Also, combined therapies employing checkpoint blockade with CAR T therapy proved to be effective (111). One the other hand, the stimulatory factors can be utilized for CAR T-cell activation. T-cells redirected for universal cytokine-mediated killing (TRUCK) are thus established and eradicate ovarian tumors *in vivo* (112).

## Controlling CAR T-cell epigenetics changes

As stem cells of hematopoietic system dividing and differentiating throughout the life, transcriptional regulators play an essential role to sustain various functions of daughter cells. CAR T-cells faces many stresses on the way to kill solid tumor cells, like persistent antigen exposure and inhibitory signals which promote the expression of exhaustion related genes such as LAG-3, TIM3, PD-1 (113). With the ongoing study of T-cell exhaustion, many evidences indicate the epigenetic changes are the major players and through manipulation of these changes T-cell can be more persistent. Direct down regulation of LAG-3, TIM-3, and PD-1 showed epigenetic changes in CAR T-cells which had enhanced tumor infiltration activity and CD56 expression, a marker of immune cell activation or cytotoxicity as well as cytokine secretion (113, 114). On the other hand, fast and continuous activation of CAR T-cells reduces pool of memory cells and results that the treatment could not last for long time (76). SOX4 and ID3 are two important factors involved in CAR T-cell exhaustion changes after long term of continuous antigen exposure. Knockout either of the two genes maintains the functional phenotypes by epigenetic modification (73). Another famous epigenetic modifier, SWI/SNF complex is related to many cancer types (115). cBAF is the most canonical sub complex of SWI/SNF and high cBAF activity determines the CD8^+^ T-cells to effector cells with decrease of memory T-cells (116, 117). Thus, cBAF activity deficient CAR T-cells improved therapy response in solid tumor models (117). Methylation of plasticity–associated genes also play a key role in T-cell dysfunction (118). Knockout DNA methyltransferase 3 alpha (DNMT3A) retained a stem-like epigenetic program in CAR T-cells and sustained cytokine expression during repeat antigen exposure (118). TET2 is another chromatin modifier that mainly activates gene expression and it is reported to activate T-cell proliferation (119). Disruption of TET2 promotes the formation of memory cells and altered T-cell differentiation that results in increased efficacy (120). TOX and TOX2 are two HMG-box transcription factors whose high expression are reported to enhance T-cell inhibitory receptors expression and their knockout greatly improved CD8^+^ T-cell function (121, 122). Like TOX, NR4A is a kind of NFAT response transcription factors including NR4A1, NR4A2 and NR4A3 with positive correlation to PD-1 and TIM3 expression. NR4A triple-knockout CAR T-cells promote tumor regression and prolong survival (123). Also, PI3Kδ/γ inhibitors help balancing CD4/CD8 ratios and increase CD8^+^ T-stem cell memory feature during CAR T-cell manufacturing process (124). In another way, CAR T-cells overexpressing c-Jun have the abilities to proliferate for long-term without exhaustion markers both *in vivo* and *in vitro* (125). One group combined the CAR with inducible activation of MyD88 and CD40 signaling pathways and showed their superior function *in vivo* and this method can be applied to other CAR T-cell enhancing factors like c-Jun activation CARs (126). All these reports suggest targeting epigenetic factors can prevent T-cell exhaustion and benefit CAR T-cell efficacy in solid tumors.

## Choosing the suitable chassis for CAR T-cell production

CAR T-cell manufacture process is highly cost and may get failure sometimes because the T-cell activity and proliferation ability from the patients are viable which can result in poor function CAR T-cells or insufficient cell amount for solid tumor treatment (26, 127). Thus, plenty of research groups turn to develop stem cells beyond PBMC derived T-cells. Induced pluripotent stem cells (iPSCs) technology exhibits great power to make large-scaled less differentiated cells through reprogramming-factors cocktail, which was discovered by Shinya Yamanaka in 2006 (128). iPSCs derived T-cells are abundant source for CAR T therapies and differentiation processes are controllable. Groups established convenient method for conventional αβT-cell production (129–131). However, the process of iPSCs to T-cells are relative time consuming (about 40 days). The αβT-iPSCs have more possibility to differentiated to CD8^+^ T-cells rather than CD4^+^ T-cells (131, 132). Highly potential proliferate T-iPSCs could cause spontaneous cancer which makes the balance between safety and efficiency a dilemma. There is still a long way to go before the CAR T-iPSCs come into solid tumor treatment.

CD4^+^ and CD8+ αβT-cells are regarded as the directly antitumor population during tumor regression but these cells separated from patients may have disordered cytotoxicity (133). Moreover, innate T-cells including NK T-cells, mucosa-associated invariant T-cells (MAITs) and γδT-cells have shown promising results for CAR-engineered therapy for cancer. These cells are broadly presented in the tumor microenvironment and their antitumor abilities are characterized long ago (134–138). With the persistence in tumor microenvironment, innate T-cells become an ideal alternative cell chassis for CAR T-cells. Human NK T-cells are a mixed group of cells containing two major populations: type I NK T-cells bearing the Vα14Jα18 invariant TCR α-chain recognizes the glycosphingolipid α-galactosylceramide (α-GalCer) and analogues through CD1d molecules and type II NK T-cells are recognizing non-α-GalCer molecules but not well defined. Type I NK T-cells, also called invariant NK T-cells, mediated the secretion of IFN-γ and TNF-α that improves antitumor effect and IL-4, IL-10, and IL-13 modulating tumor niche (139). In recent works, Andras Heczey et al. developed anti-GD2 CAR-NK T-cells, which showed great tumor infiltration ability and expansion potential that mediated tumor regression response without dose-limiting toxicities (140). In hepatocellular carcinoma, Glypican-3-specific CAR-NK T-cells also mediated potent antitumor activity while the chondroitin sulfate proteoglycan 4 (CSPG4) CAR-NK T-cells demonstrated the efficacy against melanoma (141, 142). In addition to the CAR-NK T-cells, MAIT is another rise star for CAR T-cells, which expresses the Vα7.2–Jα33 invariant TCR α-chain in humans and can penetrate into solid tumors (132). Mikail Dogan et al. generated anti-Her2 CAR-MAIT that exhibited highly activity against breast tumors and also B cell lymphoma (143). Besides, γδT-cells are featured by the γδT-cell receptors that are not MHC restrict and thus could be used to treat solid tumor with less graft-versus-host disease (GvHD) risk. Also, they have the ability to mediate fast antigen-independent cytotoxicity which reduces the escape of heterogenous tumor cells. On the other hand, the cytokine released by innate γδT-cells, especially the low level of IL-6, are different from αβT-cells and less CSR side effects (139, 144). Due to the chemokine receptors on innate αβT-cells, they can find their way into solid tumors and complete highly efficient killing. GD2-targeting CAR γδT-cells are developed and retained antigen-specific neuroblastoma cells killing as well as low exhaustion phenotypes. Nevertheless, innate γδT-cells only account for 1-5% total CD3^+^ cells, which gives rise to the short of resource during producing CAR γδT-cells (145).

Immune system is a well-organized pool of different cells, in which each type of cells contributes to the overall function. The significance of crosstalk between multiple T-cells suggests that mixed population can achieve better anti-tumor efficacy (146–148). In summary, further knowledge about the function of different populations and study of the optimal T-cells combination may lead to the highly efficient CAR T-system rather than single kind CAR T-cells.

## Utilizing combined therapies

**Figure 3:**
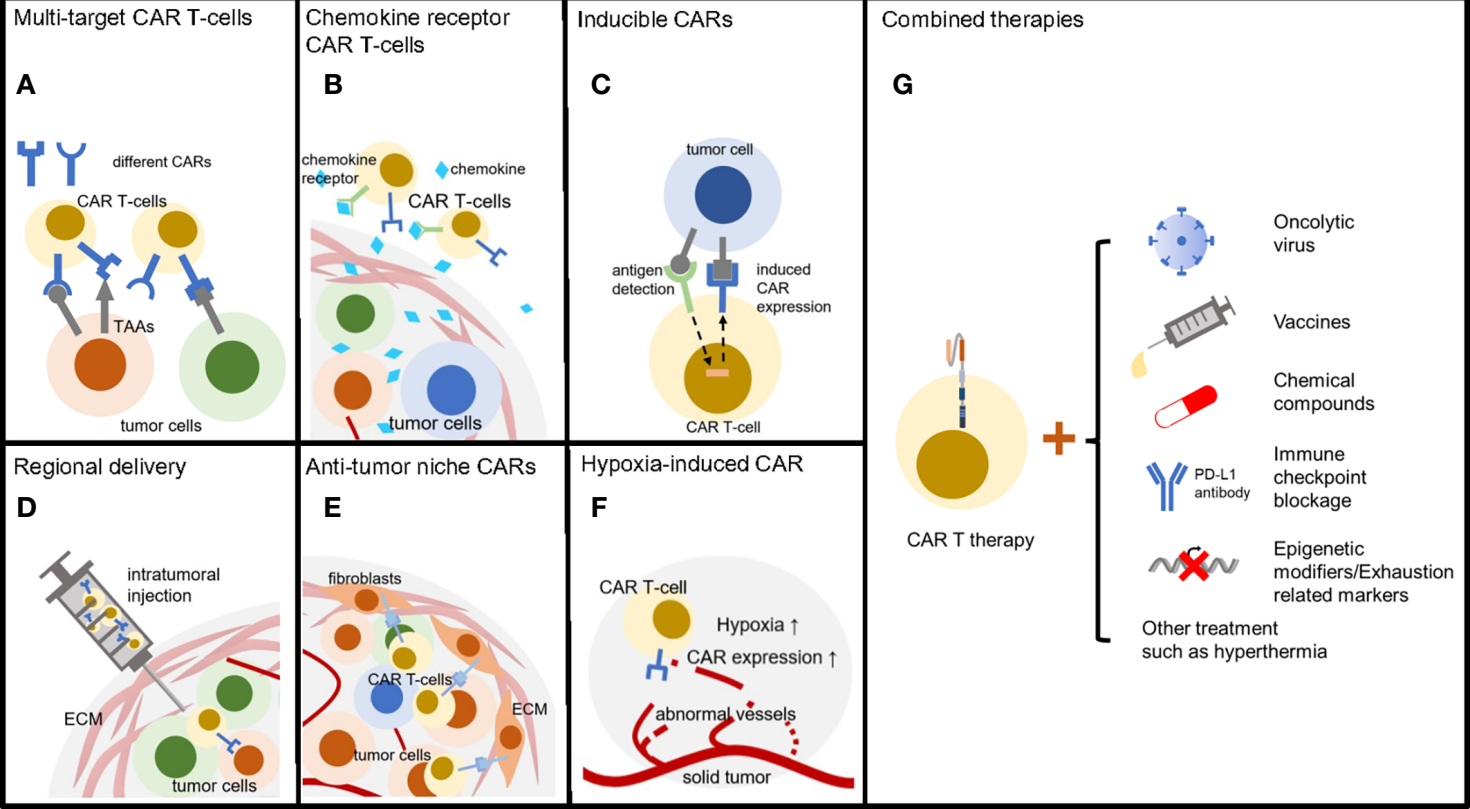
Methods to reverse CAR T therapy resistance. **(A)** T-cells armored with multi-specific CARs can broadly target heterogeneous tumor cells. **(B)** CAR T-cells with chemokine receptor are able to efficiently infiltrate solid tumors. **(C)** Inducible CARs enable T-cells precisely kill tumors cell with low exhaustion and off-tumor cytotoxicity. **(D)** Regional delivery directly transfers the CAR T-cells into solid tumors. **(E)** CARs targeting tumor niche can be an alternative anti-cancer choice through destroying of microenvironment. **(F)** Hypoxia inducible CARs have low off-target effect due to the regional activation. **(G)** Combined therapies including oncolytic virus, tumor vaccines, chemical drugs, ICB and epigenetic modifiers enhanced CAR T-cell treatment efficacy.

Solid tumors have many approaches to escape anti-tumor therapies and therefore by no means we only take one treatment in the case that no obvious side effect is detected. Combined therapies can take out additive effect, synergistic effect or even synthetic lethality (149). Lots of clinical trials are going on to check out the proper secondary treatment with CAR T-cell therapies (**Figure 3**). Growing quantity of new combinations are now reported with improving immune attack of solid tumors.

As mentioned above, solid tumors have few proper tumor-specific antigens to attract CAR T-cells. But oncolytic viruses can modify the tumor microenvironment and recruit activated T-cells. VSV-mIFNβ can promote a favorable chemokine profile for CAR T-cells and stimulate virus specific TCR activity which boosts memory CAR T-cells function and proliferation (150). This leads to better survival of subcutaneous melanoma and intracranial glioma tumors bearing mice (150). A study from the same group noticed the importance of administration schedule. Pre-treated with VSV-mIFNβ followed by CAR T-cell injection would induce virus-derived type I interferon that caused CAR T-cell death (151). Leyuan Ma et al. designed a kind of vaccine called amph-ligand that can be processed and presented on APCs (152). The amph-ligand contains a CAR ligand domain and CAR T-cells interacting with the APCs will be stimulated and expanses. This strategy is easy to apply to all CAR T-cell therapies and overcomes the low antigen expression of solid tumors. Tumor infiltrated CAR T-cells are ideal carriers of drug. Synthetic enzyme-armed killer (SEAKER) cells process small-molecule prodrug within the tumor and release the active drug such as 5’-O-Sulfamoyladenosine (AMS) resulting in strong antitumor response (153). It is known that mild hyperthermia can reduce solid tumor compact structure and promote immune cell infiltration. Recent study demonstrated photothermal ablation of the tumor can be combined with CAR T-cell therapies to maximize antitumor activity of melanoma (154). Solid tumors often ware abnormal glycosylation to mask the epitope of antigens and interfere with antitumor immunity (155, 156). One group recently combined 2-deoxy-D-glucose (2DG) treatment, a kind of glucose/mannose analog that can inhibit glycosylation, with CAR T-cell therapy and obtained promising efficacy against multiple solid carcinomas (157).

Checkpoint blockade and CAR T therapy co-treatment is another effective combined therapy considering the immunosuppressive tumor niche. Tumor infiltrated CAR T-cells are highly possible to express signs of phenotypic exhaustion such as PD-1 and reasonably blocking checkpoint could protect the CAR T-cells from immunosuppressive microenvironment (**Figure 3** and **Table 1**) (111, 158, 159). Recent studies demonstrated the potential in both ovarian cancer and lung cancers. Additionally, the hydrogel containing CAR T-cells and anti-PD-L1-conjugated platelets developed by Quanyin Hu et al. provided a state-of-the-art way for solid tumor treatment (159). And now several clinical trials are going to study the efficacy of combined Checkpoint blockade and CAR T combined therapies.

Despite the existing therapeutic pairs, new druggable targets are rising. Robert T. Manguso et al. discovered a powerful immunotherapy target called phosphatase nonreceptor type 2 (PTPN2) through *in vivo* CRISPR screening (9). Deletion of PTPN2 results in enhanced T-cell stimulation and infiltration. Despite the tumor modification function, PTPN2 knockout also increased proliferative capacity of CD8^+^ T-cells (160). Thus, PTPN2 is suggested to be a potent mate with CAR T-cell therapies in solid tumors and it is valuable to develop PTPN2 inhibitors. PTPN22 is another immune modulator related to T-cell function (12, 161). Its activation plays a negative role in immune responses and thus inhibition of PTPN 22 may enhance antitumor response (8). However, it is controversial about how to combine this target with CAR T-cell therapies. PTPN 22 knockout only in CAR T-cells could not improve the efficacy but when systemically inhibited, it augments tumor repressing function (162). And one group has developed a PTPN22 inhibitor which greatly promotes anticancer immunity (8).

Though some combined therapies may be suitable for both hematological malignancy and solid tumors, we should be careful about the intrinsic differences between them. IFN-gamma signaling is well known for its importance to control tumor growth and mutation of IFN-gamma related component can result in resistance of immune therapies (163). One group compared the IFN-gamma signaling in solid and liquid tumors and found only killing solid tumors required IFN-gamma pathway (164). This result indicates the combination of IFN-gamma with CAR T therapies may not benefit blood tumor treatment. Accordingly, some promising targets combined with CAR T therapies in blood cancer could have no positive results in solid cancer due to their distinct reliance, which emphasizes more thorough researches and preclinical test.

## Conclusions

After many years’ attempt, many researchers finally turn the concept of CAR T-cells into a powerful adoptive cell therapy and it acquired so much success in the past few years especially for blood malignancy. Advances learned from hematologic tumors treatment equip CAR T-cells with new-generation armors but there are still lots of considerations due to the complexity of the solid tumor itself, the microenvironment and immune system. Luckily, more and more fantastic ideas are placed into practice including CAR modifications, T-cell enhancement and collaborative therapies. These methods provided positive cases in many solid tumor models and even in patients. Beside the evolution on CAR T-cells, the differences between blood and solid tumors are getting clear and discovery of pan-immune system modulators such as PTPN family members strengthens the application of CAR T therapies. In short, CAR T-cells have great potential to be easily modified according to rising concepts to reverse specific tumor resistance. However, the remaining problem is how to optimize CAR T-cells through integrating the advantages of existing and new-born methods, which needs more research practice and clinical investigations.

On the other hand, few studies discuss the challenges of CAR T therapy during the CAR manufacture process and the component of CAR T-cells may also play an important role during solid tumor treatment such as the ratio of different T-cells. Furthermore, CAR T therapies induced side effect is a barrier restricting the effective cell dose during solid tumor treatment. Larger cell dose means both stronger opposite and side effect, so it is also valuable to explore the proper methods to reduce off-tumor effect. In all, CAR T-cells treating solid tumors are promising with the continuous efforts on the bench and in the hospital.

## Author contributions

GX and YQ conceptualized the frame work. YQ wrote the manuscript and made the figures under the supervision of GX. YQ and GX contributed to the editing of the content. All authors reviewed the article and agreed to the submitted version.
